# Dihydroartemisinin Protects against Dextran Sulfate Sodium-Induced Colitis in Mice through Inhibiting the PI3K/AKT and NF-*κ*B Signaling Pathways

**DOI:** 10.1155/2019/1415809

**Published:** 2019-11-06

**Authors:** Ning Li, Wenjing Sun, Xin Zhou, Hao Gong, Yuqing Chen, Dongfeng Chen, Fei Xiang

**Affiliations:** ^1^Department of Gastroenterology, Daping Hospital, Army Medical University, Chongqing 400030, China; ^2^Institute of Burn Research, State Key Laboratory of Trauma, Burns and Combined Injury, Southwest Hospital, Third Military Medical University (Army Medical University), Chongqing 400038, China

## Abstract

Ulcerative colitis is a common inflammatory bowel disease, and the activation of thePI3K/AKT and NF-*κ*B signaling pathways plays a pivotal role in its pathogenesis. Dihydroartemisinin (DHA) is a widely used antimalarial drug and has shown anticancer effect partially through inhibiting the activation of PI3K/AKT and NF-*κ*B. This study aimed to investigate the effect of dihydroartemisinin on ulcerative colitis and its mechanism. Adult male C57 mice were subjected to 3.0% dextran sulfate sodium (DSS) for seven days; simultaneously, dihydroartemisinin or control saline was administered by oral gavage once a day. In vitro, the intestinal epithelial cell-6 was treated with LPS for 24 hours with or without dihydroartemisinin combined with PI3K/Akt activator 740 Y-P or NF-*κ*B activator phorbol myristate acetate. Western blotting was used to test the activation of PI3K/AKT and NF-*κ*B. Dihydroartemisinin significantly ameliorated body weight loss, shortened colon length, and increased DAI in DSS-induced colitis. Meanwhile, histological damage was improved and was accompanied by decreased expression and secretion of proinflammatory cytokines. Moreover, DSS-induced elevation of phosphorylation of PI3K, AKT, IKK*α*, I*κ*B*α*, and NF-*κ*B (p65) was remarkably blunted by dihydroartemisinin both in vivo and in vitro, indicating an inhibitive property on the PI3K/AKT and NF-*κ*B signaling pathways. Furthermore, administration of 740 Y-P or PMA significantly blocked protective activity of dihydroartemisinin against colitis in vitro. In conclusion, dihydroartemisinin can attenuate DSS-induced colitis, and its anticolitis effect might be mediated via the PI3K/AKT and NF-*κ*B signaling pathways. DHA might serve as a promising drug for patients with ulcerative colitis.

## 1. Introduction

Ulcerative colitis (UC) is an inflammatory bowel disease (IBD) caused by a combination of environmental and genetic factors and is characterized by inflammation, homeostasis disruption, and epithelial barrier damage in the intestinal tract [1, 2]. UC affects millions of people worldwide, and its morbidity increases yearly. The clinical manifestations of UC include development of bloody diarrhea with or without mucus, weight loss, abdominal pain, rectal urgency, tenesmus, and even systemic symptoms in severe cases [3]. Currently, the main therapy for UC is anti-inflammatory treatment using nonsteroid drugs such as mesalamine and corticosteroids. However, the nonsteroid drugs lack a universal response and have potential adverse side effects, such as infection and neoplasia. Therefore, it is urgent to develop new drugs for safer and more effective treatment of UC.

So far, although the pathophysiology of UC is not fully understood, promising advances have been achieved in the knowledge of inflammatory and immune mechanisms [4]. Many cellular signaling pathways, such as MAPK/AP-1, Nrf2, PPAR-*α*, PI3K/AKT, and NF-*κ*B, are generally believed to be involved in inflammatory and immune disorders in UC. For example, rSj16 can inactivate the PPAR-*α* signaling pathway to inhibit immune inflammatory responses and protect against DSS-induced colitis [5]. LL202 protects against experimental colitis in mice by inhibiting MAPK/AP-1 signaling to decrease the expression of proinflammatory factors [6]. Notably, PI3K/AKT and NF-*κ*B signaling pathways are appealing for their crucial role in pathogenesis and progress of UC and are regarded as an important target for UC treatment. The PI3K/AKT signaling pathway is involved in release of proinflammatory cytokines, such as TNF-*α* in the intestinal mucosa in UC patients, and targeting this pathway can provide the basis for development of new drugs for UC [7, 8]. Activation of the NF-*κ*B signaling pathway plays a central role in regulating an immune inflammatory response in UC, and there is evidence that inactivation of NF-*κ*B can attenuate experimental colitis [4, 9].

Dihydroartemisinin (DHA) is an artemisinin derivative and is widely used as a first-line antimalarial drug. Additionally, growing evidence has shown that dihydroartemisinin exerts beneficial effects in many diseases, especially in cancer [10–12]. For example, dihydroartemisinin can attenuate hepatocyte lipoapoptosis and liver fibrosis [13, 14]. Dihydroartemisinin also inhibits the growth of squamous cell carcinoma cells and colorectal carcinoma cells [10]. These effects were partially mediated by its inhibitive property on the PI3K/AKT and NF-*κ*B signaling pathways [15–17]. However, whether dihydroartemisinin exerts a protective effect against UC is unknown. In the present study, we tested the effect of dihydroartemisinin on DSS-induced colitis and found that it significantly attenuated DSS-induced colitis and inhibited the expression of proinflammatory factors. We also revealed that dihydroartemisinin played an anticolitis role through inhibiting the PI3K/AKT and NF-*κ*B signaling pathways.

## 2. Materials and Methods

### 2.1. Induction and Evaluation of Colitis in Mice

Four-month-old C57BL/6J male mice were obtained from the Laboratory Animal Center of Daping Hospital. All experimental procedures were in accordance with institutional regulations and approved by the Experimental Animals Committee of Daping Hospital. Animals were randomly allocated into three groups: control group (*N* = 8), DSS group (*N* = 8), and DSS + DHA group (*N* = 8 for each dose). Colitis was induced by replacing drinking water with 3.5% DSS (MP Biomedicals, USA) for seven days. Meanwhile, DHA was administered by oral gavage once a day for 7 days. According to a previous study, dose of dihydroartemisinin varying from 10 mg/kg d to 50 mg/kg d has been widely used in many animal models such as cancer and it showed great efficacy and safety [18–21]. Therefore, doses of 10, 25, and 50 mg/kg d were selected in the present study to test dihydroartemisinin's protective effect against DSS-induced colitis. Body weight loss, stool consistency, and fecal blood loss were observed and recorded daily. The clinical course of colitis was evaluated by a daily disease activity index consisting of the three parameters, stool consistency, weight loss, and the presence of blood in feces and the anus, as previously described [22]. At day 7, animals were euthanized with an overdose of isoflurane and the colon and blood samples were collected. Then, colon length was measured, and the colon was stored for subsequent experiments.

### 2.2. Histopathology Analysis

Histopathology of the colon was examined using hematoxylin-eosin (H&E) staining as previously described [23]. Briefly, approximately 0.5 cm of the descending colon was obtained and cleaned with normal saline. Then, samples were fixed with 4% paraformaldehyde, dehydrated, and paraffin embedded prior to being sectioned continuously at a thickness of 4 *μ*m. The colon sections were rehydrated, stained with hematoxylin and eosin, and mounted in permount. Then, the sections were observed with a microscope to observe morphological changes. Histopathological scores of the colonic lesions were determined as previously described by evaluation of inflammation, crypt damage, submucosal edema, and reactive epithelial hyperplasia [5].

### 2.3. ELISA Analysis

Colon homogenate supernatants and serum were collected, and the concentrations of TNF-*α*, IL-6, and IL-1*β* were measured by using ELISA kits following the manufacturer's instructions (NeoBioscience, China).

### 2.4. Cell Culture

Intestinal epithelial cell-6 (IEC-6) was cultured in DMEM medium and was treated with LPS (1 *μ*g/ml) for 24 hours to establish in vitro colitis model. Dihydroartemisinin (100 *μ*M) with/without PI3K/Akt activator 740 Y-P (25 *μ*g/mL) or NF-*κ*B activator phorbol myristate acetate (PMA, 1 *μ*M) was administrated for 24 hours. Then, cells were collected and stored at −80°C for western blotting and quantitative real-time PCR.

### 2.5. Quantitative Real-Time PCR

Total RNA was isolated from the colon tissue or IEC-6 cells using RNAiso plus (Takara, Japan) following the manufacturer's instructions, and reverse transcription polymerase chain reaction (RT-PCR) was performed using the TOYOBO RT-PCR kit. The Applied Bio systems 7500 Fast Real-Time PCR System was used to perform SYBR Green quantitative PCR of TNF-*α*, IL-6, and IL-1*β*. Quantification of mRNA expression was normalized against GAPDH using the double delta Cq method. The primers used are listed in Table 1.

### 2.6. Western Blotting Analysis

The activation of the PI3K/AKT and NF-*κ*B signaling pathways in colon tissue was measured by western blotting. The total protein of colon tissues was extracted using RIPA lysis buffer containing a protease inhibitor cocktail (Beyotime Biotechnology, China), and protein concentrations were measured using a Bradford protein assay kit (Bio-Rad, USA). The proteins were separated by SDS-PAGE and then electrotransferred to PVDF membranes. The membranes were then blocked with 5% nonfat skim milk and incubated with primary antibodies overnight at 4°C. Then, membranes were washed with TBST and incubated with secondary antibody (goat anti-rabbit IR Dye 800, Invitrogen) for 1 hour at room temperature, followed by visualization with an Odyssey Infrared Imaging System (Li-Cor Biosciences, Lincoln, NE) and analysis using Quantity One image analysis software. GAPDH was used as an internal control to normalize the expression of protein. The antibodies used were as follows: PI3K (1 : 1000; Cell Signaling Technology), phosphor-PI3K (1 : 1000; Cell Signaling Technology), AKT (1 : 1000; Abcam), p-AKT (1 : 800; Abcam), NF-*κ*Bp65 (1 : 800; Abcam), p-I*κ*B*α* (1 : 800; Abcam), I*κ*B*α* (1 : 800; Abcam), IKK*α* (1 : 800; Abcam), p-IKK*α* (1 : 800; Abcam), and GAPDH (1 : 1000; Cell Signaling Technology).

### 2.7. Statistical Analysis

All results are expressed as means ± SD. Statistical analysis was performed using SPSS 18.0 software. Comparison of means between two groups was performed using the two-tailed Student's *t*-test. Comparison of means among more than two groups was performed by one-way ANOVA followed by Holm–Sidak's post hoc multiple comparison test. *p* < 0.05 was considered statistically significant.

## 3. Results

### 3.1. Dihydroartemisinin Ameliorated DSS-Induced Colitis in Mice

**Figure 1:**
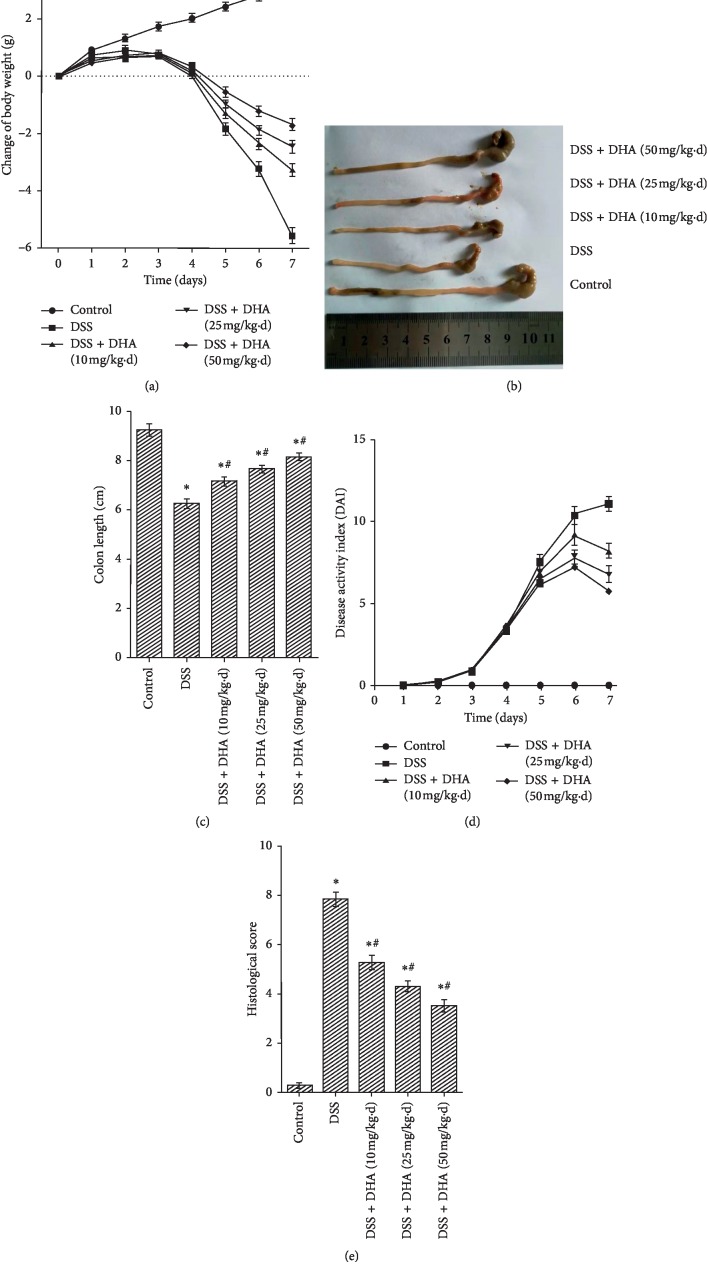
Dihydroartemisinin protects against DSS-induced colitis in mice. (a) Changes in body weight were recorded daily. (b) Representative images of colons on day 7. (c) Quantification of colon lengths on day 7. *N* = 8 in each group. (d) The disease activity index (DAI) of mice on day 7. *N* = 8 in each group. (e): Histological scores were assessed to evaluate macroscopic colon damage. *N* = 8 in each group. ^*∗*^*p* < 0.05 vs. control group. ^#^*p* < 0.05 vs. DSS group.

To explore whether dihydroartemisinin exerts therapeutic effects on colitis, we established a mouse model of DSS-induced colitis and dihydroartemisinin was administered daily at various doses by oral gavage for 7 days. The results showed that DSS induced drastic body weight loss, and dihydroartemisinin administration significantly blunted the body weight loss in a dose-dependent manner (Figure 1(a)). In addition, DSS induced a significant shortening of the colon length (Figures 1(b) and 1(c)), which is a marker of sufficient induction of colitis and is inversely associated with the severity of colitis. Consistent with attenuated body weight loss, the shortening of the colon length was significantly improved by dihydroartemisinin in a dose-dependent manner (Figures 1(b) and 1(c)). To further investigate the effect of dihydroartemisinin on DSS-induced colitis, evaluation of DAI (disease activity index) was performed. As shown in Figure 1(d), DSS induced a DAI increase during disease progression, and this elevation was relieved by dihydroartemisinin from day 4 onward. Macroscopic analysis of the colon revealed that DSS induced a significant increase in the macroscopic colon damage scores which was characterized by hyperemia, ulceration, and bowel wall thickening (Figure 1(e)). Notably, macroscopic colon damage scores in the dihydroartemisinin group were significantly lower than those in the DSS group, indicating improvement of colon damage (Figure 1(e)).

### 3.2. Dihydroartemisinin Reduced Microscopic Colon Damage in DSS-Induced Colitis

**Figure 2:**
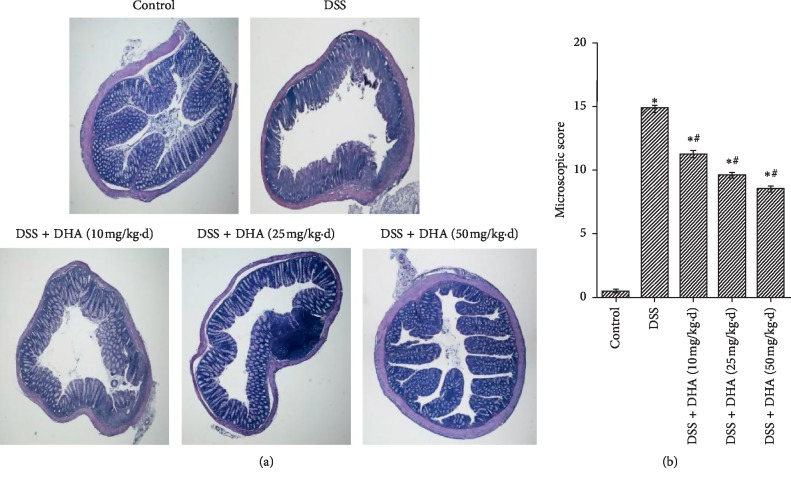
Dihydroartemisinin reduces microscopic colon damage in DSS-induced colitis. (a) Representative images of colonic tissue sections with hematoxylin & eosin (H&E) staining. (b) Histopathological scores were determined. *N* = 8 in each group. ^*∗*^*p* < 0.05 vs. control group. ^#^*p* < 0.05 vs. DSS group.

To further investigate the histological changes in the colons, H&E staining was carried out. Our results showed that compared with the control group, the colon specimens in the DSS group displayed severe mucosal damage, distortion of crypts, infiltration of mononuclear cells, and loss of goblet cells, confirming that the model of DSS-induced colitis in mice was successfully established (Figure 2(a)). Consistent with the observed anticolitis effect in Figure 1, dihydroartemisinin largely restored DSS-induced histopathological abnormities, which was further confirmed by microscopic scores (Figures 2(a) and 2(b)). Collectively, these results suggested that dihydroartemisinin can reduce microscopic colon damage, consistent with its therapeutic effect in colitis.

### 3.3. Dihydroartemisinin Inhibited Production of Proinflammatory Cytokines in DSS-Induced Colitis

**Figure 3:**
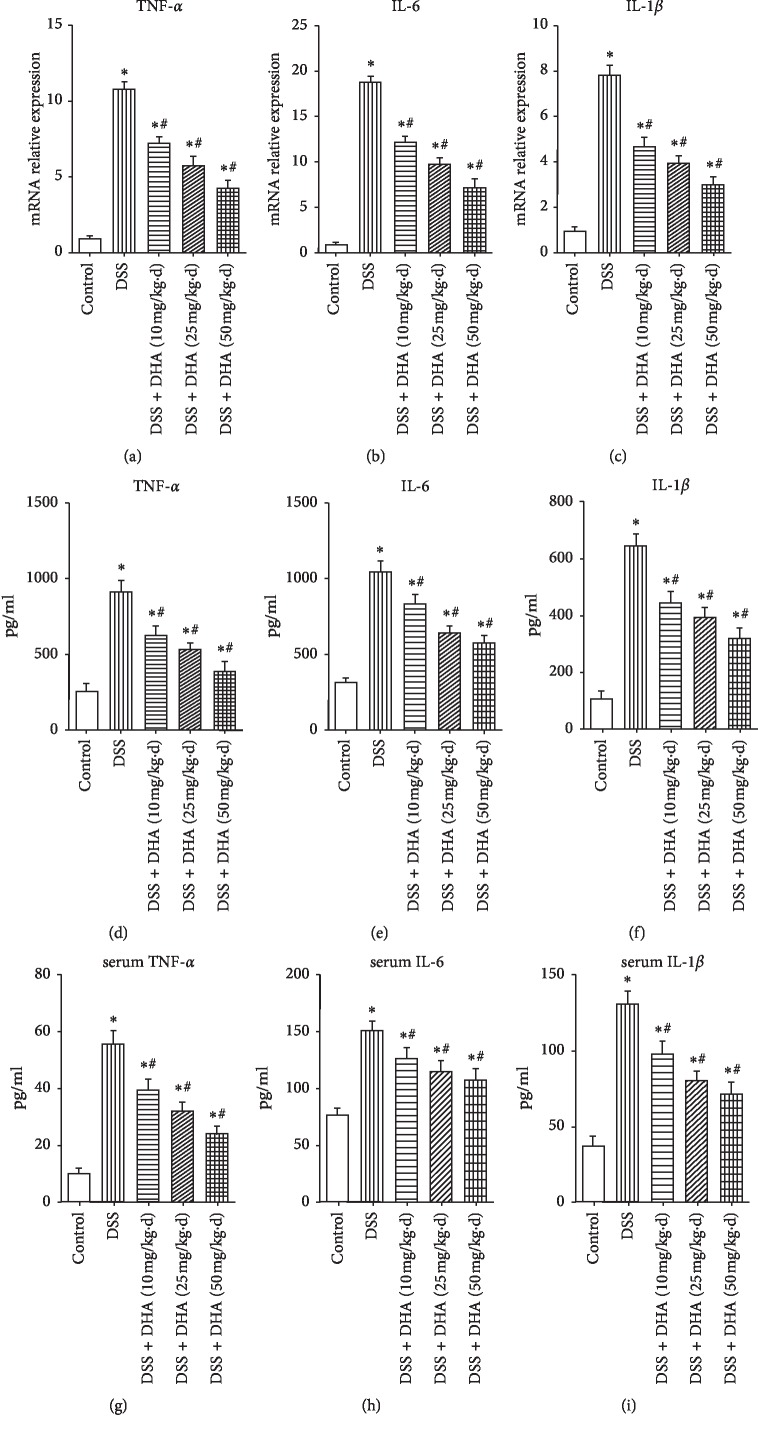
Dihydroartemisinin inhibits production of proinflammatory cytokines in the colon. (a–c) mRNA levels of TNF-*α*, IL-6, and IL-1*β* in colonic homogenates determined by qRT-PCR. *N* = 4 in each group. (d–f) Protein levels of TNF-*α*, IL-6, and IL-1*β* in colonic homogenates determined by ELISA. *N* = 5 in each group. (g–i): Serum concentrations of TNF-*α*, IL-6, and IL-1*β* determined by ELISA. *N* = 6 in each group. ^*∗*^*p* < 0.05 vs. control group. ^#^*p* < 0.05 vs. DSS group.

As proinflammatory factors play important roles in the pathogenesis and progress of UC [24], we investigated whether dihydroartemisinin inhibits production of proinflammatory cytokines in the colon and serum. As shown in Figures 3(a)–3(c), DSS induced a significant increase in mRNA expression of TNF-*α*, IL-6, and IL-1*β*, and these changes were blocked by dihydroartemisinin in a dose-dependent manner, indicating that the transcription of these cytokines was inhibited by dihydroartemisinin. In addition, ELISA analysis of the colonic homogenized protein revealed that the protein levels of TNF-*α*, IL-6, and IL-1*β* were remarkably increased in the DSS group compared to those in the control group (Figures 3(d)–3(f)). Similarly, administration of dihydroartemisinin inhibited the production of these cytokines in a dose-dependent manner (Figures 3(d)–3(f)). Furthermore, similar alterations of serum TNF-*α*, IL-6, and IL-1*β* were observed (Figures 3(g)–3(i)). These data suggested that dihydroartemisinin exerts an anti-inflammatory effect in colitis, and this effect may account for its therapeutic role in DSS-induced colitis.

### 3.4. Dihydroartemisinin Blocked the Activation of PI3K/AKT and NF-*κ*B Signaling Pathways in Colitis Both In Vivo and In Vitro

**Figure 4:**
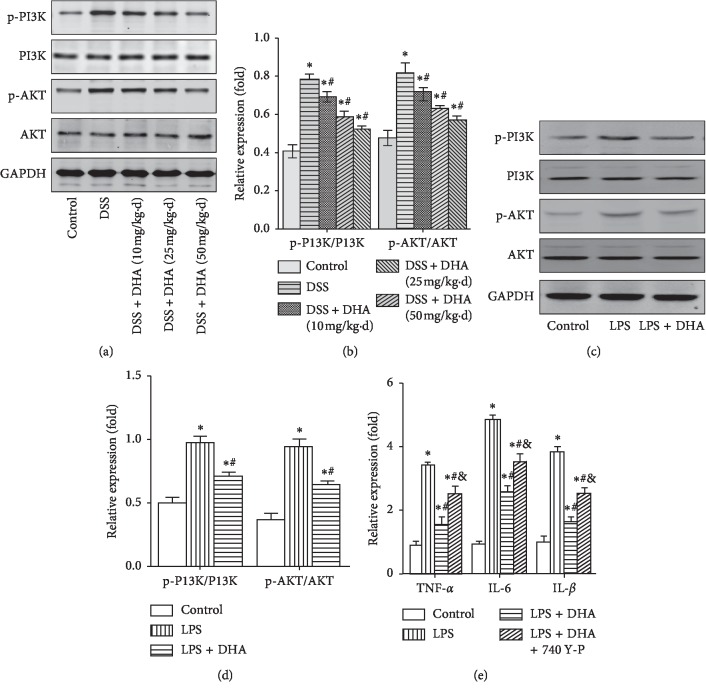
Dihydroartemisinin blocks activation of the PI3K/AKT signaling pathway in colitis both in vivo and in vitro. The mice were treated with DSS for 7 days and IEC-6 cells were treated with LPS for 24 hours, and then, the colon tissue and IEC-6 cell lysates were analyzed by western blotting. (a): Representative image of p-PI3K, PI3K, p-AKT, and AKT in colon tissues. (b): Quantification analysis of p-PI3K/PI3K and p-AKT/AKT in colon tissues. *N* = 4 in each group. ^*∗*^*p* < 0.05 vs. control group. ^#^*p* < 0.05 vs. DSS group. (c) Representative image of p-PI3K, PI3K, p-AKT, and AKT in IEC-6 cells. (d) Quantification analysis of p-PI3K/PI3K and p-AKT/AKT in IEC-6 cells. *N* = 4 in each group. (e) Relative mRNA expression of TNF-*α*, IL-6, and IL-1*β* in IEC-6 cells. *N* = 4 in each group. ^*∗*^*p* < 0.05 vs. control group; ^#^*p* < 0.05 vs. LPS group; and *p* < 0.05 vs. LPS + DHA group.

Since the PI3K/AKT and NF-*κ*B signaling pathways play essential roles in colitis [25–28], we next determined the effect of dihydroartemisinin on the PI3K/AKT and NF-*κ*B signaling pathways in colitis both in vivo and in vitro. Western blotting results showed that DSS treatment increased the level of p-PI3K and p-AKT and the relative level of p-PI3K/PI3K and p-AKT/AKT in colon tissue, indicating the activation of PI3K/AKT cascades (Figures 4(a) and 4(b)). Notably, administration of dihydroartemisinin largely blocked the activation of the PI3K/AKT signaling pathway, which was indicated by lower p-PI3K/PI3K and p-AKT/AKT (Figures 4(a) and 4(b)). Similarly, LPS treatment increased the level of p-PI3K and p-AKT and the relative level of p-PI3K/PI3K and p-AKT/AKT in cultured IEC-6 cells, and these alterations were significantly ameliorated by dihydroartemisinin (Figures 4(c) and 4(d)). Furthermore, dihydroartemisinin blocked LPS-induced upregulation of TNF-*α*, IL-6, and IL-1*β* in IEC-6 cells, and this effect was partially blocked by administration of PI3K/AKT activator 740 Y-P (Figure 4(e)), demonstrating that dihydroartemisinin protects against colitis partially through inhibiting activation of PI3K/AKT signaling pathway.

**Figure 5:**
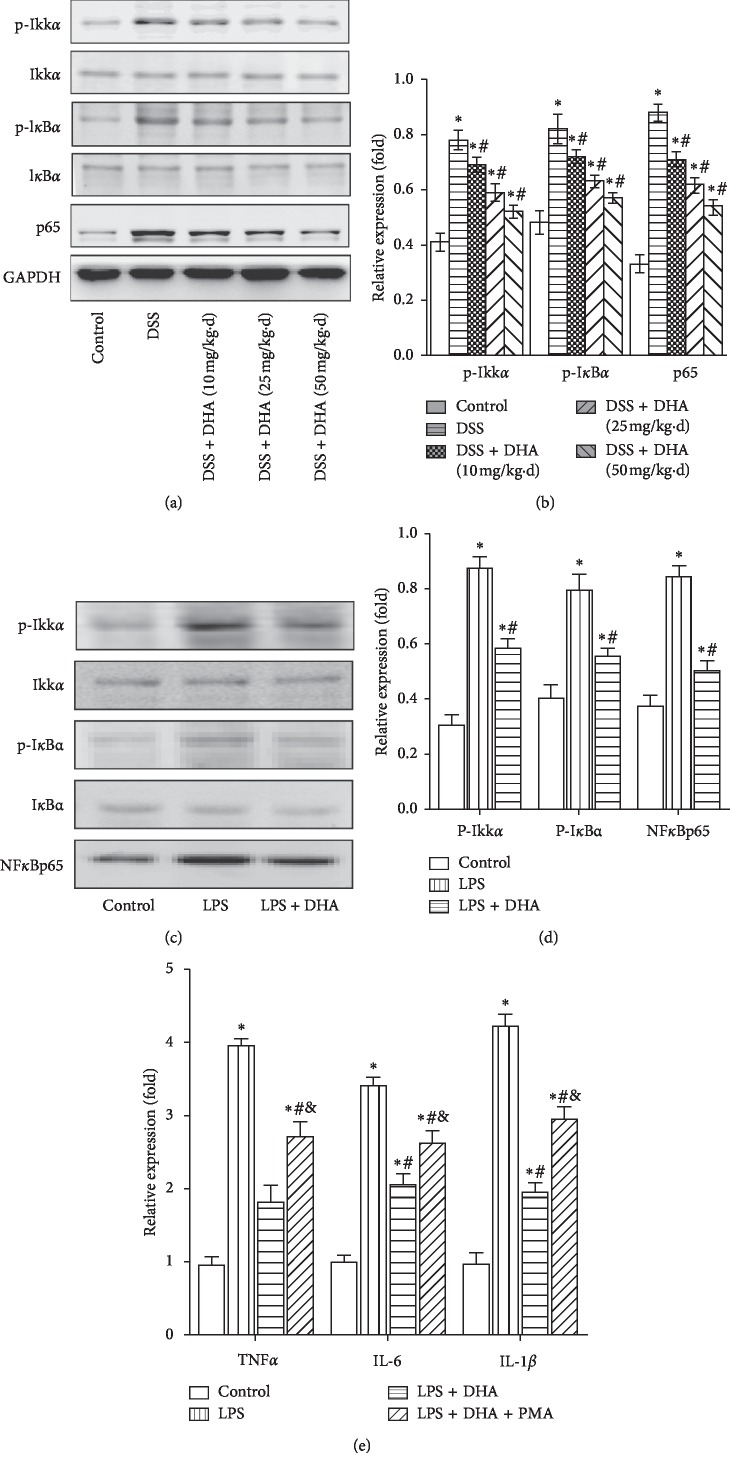
Dihydroartemisinin blocks activation of the NF-kB signaling pathway in colitis both in vivo and in vitro. The mice were treated with DSS for 7 days, and IEC-6 cells were treated with LPS for 24 hours, and then, the colon tissue and IEC-6 cell lysates were analyzed by western blotting. (a) Representative image of p-IKK, IKK, p-IkBa, IkBa, and NF-kB (p65) in colon tissues. (b) Quantification analysis of p-IKK/IKK, p-IkBa/IkBa, and NF-kB in colon tissues. *N* = 4 in each group. ^*∗*^*p* < 0.05 vs. control group. ^#^*p* < 0.05 vs. DSS group. (c) Representative image of p-IKK, IKK, p-IkBa, IkBa, and NF-kB (p65) in IEC-6 cells. (d) Quantification analysis of p-IKK/IKK, p-IkBa/IkBa, and NF-kB in IEC-6 cells. *N* = 4 in each group. (e) Relative mRNA expression of TNF-*α*, IL-6, and IL-1*β* in IEC-6 cells. *N* = 4 in each group. ^*∗*^*p* < 0.05 vs. control group; ^#^*p* < 0.05 vs. LPS group; & *p* < 0.05 vs. LPS + DHA group.

DSS also induced activation of the NF-*κ*B signaling pathway in colon tissue, indicated by increased phosphorylation of IKK*α*/I*κ*B*α* and NF-*κ*B (p65), which was inhibited by dihydroartemisinin (Figures 5(a) and 5(b)). In vitro results showed that LPS treatment induced elevation in phosphorylation of IKK*α*/I*κ*B*α* and NF-*κ*B (p65), and these alterations were blocked by dihydroartemisinin (Figures 5(c) and 5(d)). Protective effect of dihydroartemisinin against colitis was also observed in vitro, and this effect was partially blocked by administration of NF-*κ*B activator PMA (Figure 5(e)). Therefore, these results indicated that the PI3K/AKT and NF-*κ*B signaling pathways were activated in colitis, and dihydroartemisinin may exerts its benefits in DSS-induced colitis by inhibiting the activation of both PI3K/AKT and NF-*κ*B signaling pathways.

## 4. Discussion

In the present study, we investigated the protective effect of dihydroartemisinin on DSS-induced colitis and explored the underlying mechanism. We found that dihydroartemisinin significantly ameliorated body weight loss, shortened the colon length, and increased DAI in DSS-induced colitis. Meanwhile, histological damage was improved, accompanied by decreased expression and secretion of proinflammatory cytokines. For the mechanism, the anticolitis effect of dihydroartemisinin might be mediated by its inhibition of the PI3K/AKT and NF-*κ*B signaling pathways.

UC is characterized by inflammation, homeostasis disruption, and epithelial barrier damage in the intestinal tract, and DSS is a well-established agent to induce experimental colitis that is phenotypically similar to UC in humans [29]. DSS-induced colitis is of reasonable reproducibility and of great value for investigating the efficacy of anticolitis agents. In our study, oral administration of DSS in mice for several days induced significant body weight loss and shortening of the colon length, which was comparable to previous studies [30, 31]. In addition, evaluation of weight loss, stool consistency, and paranal bleeding revealed that DSS induced a dramatic increase in DAI score. Meanwhile, histological damage was observed in the DSS group. These results were consistent with published data in previous reports, confirming successful establishment of DSS-induced colitis. Therefore, these results make our experiments based on DSS-induced colitis reliable.

Although the mechanism of pathogenesis of UC is not yet fully understood, the abnormality of the inflammatory and immune responses has been highly focused on. Evidence from previous studies has demonstrated that interaction between the intestinal mucosal immune system and proinflammatory cytokines can affect intestinal homeostasis and lead to the disruption of tight junction proteins [32]. Disorders in immune responses have been reported to be associated with imbalance between proinflammatory and anti-inflammatory cytokines, and increased expression of proinflammatory cytokines is an important characteristic of UC [4, 29]. In our study, DSS treatment in mice remarkably induced elevation of mRNA expression of TNF-*α*, IL-6, and IL-1*β*, which was consistent with previous studies [9, 27]. In addition, the protein levels of these proinflammatory cytokines were also significantly increased after DSS treatment. Notably, dihydroartemisinin treatment dramatically suppressed the elevation of both colon and serum proinflammatory cytokines. Therefore, we speculated that the protective effect of dihydroartemisinin might be related to inhibition of inflammatory response.

Although, many intracellular signaling pathways are involved in regulating the inflammatory and immune responses, growing evidence has shown that the PI3K/AKT signal transduction pathway plays a pivotal role in regulation of expression and secretion of inflammatory cytokines. In addition, the PI3K/AKT signaling pathway is abnormally activated in UC, leading to enhanced expression and secretion of proinflammatory cytokines such as TNF-*α* [7, 22]. In addition to the PI3K/AKT signaling pathway, activation of NF-*κ*B can enhance production of proinflammatory cytokines [9, 31, 33]. In the present study, the results showed that DSS and LPS induced activation of PI3K/AKT and NF-*κ*B in vivo and in vitro, respectively, and the activation was prevented by dihydroartemisinin. Furthermore, protective effect of dihydroartemisinin against colitis was partially blocked by PI3K/Akt activator 740 Y-P or NF-*κ*B activator PMA, demonstrating that the anti-inflammatory effect in colitis might be mediated by PI3K/AKT and NF-*κ*B signaling suppression. Dihydroartemisinin has also been reported to inhibit endothelial cell proliferation through the suppression of the MAPK (mainly ERK subfamily) signaling pathway [34]. However, in another study, dihydroartemisinin was reported to activate ERK and p38 MAPK signaling pathways in PC12 cells [35]. In addition, dihydroartemisinin was also reported to suppress TGF-*β* signaling in the lung and kidney [36, 37]. Therefore, the effect of dihydroartemisinin on these signaling cascades might depend on the cell and organ type and experimental settings. As MAPK and TGF*β* signaling pathways involve in pathogenesis of colitis [6], there exists the possibility that dihydroartemisinin also partially regulates MAPK and TGF*β* pathways to exert its protective effect against colitis. In addition, there are crosstalks between these signaling pathways. For example, TGF*β* was reported to activate PI3K-AKT signaling [38], which further complicated potential regulatory network in colitis. Therefore, further studies are needed to define relationship between these signaling pathways and dihydroartemisinin in colitis.

## 5. Conclusion

Our results first revealed that dihydroartemisinin can effectively attenuate DSS-induced colitis. In addition, DSS-induced elevation of expression and secretion of proinflammatory cytokines was blocked by dihydroartemisinin. Additionally, the protective effect of dihydroartemisinin might occur through inhibition of both the PI3K/AKT and NF-*κ*B signaling pathways. Notably, dihydroartemisinin has been indicated to be safe in the treatment of malaria and application in cancers. Therefore, dihydroartemisinin might be a promising molecule that has translational potential in the treatment of colitis.

## Figures and Tables

**Table 1:** Primers for real-time PCR.

Primer name	Species	Sequence
TNF-*α* forward	Mouse	CCCTCACACTCACAAACCAC
TNF-*α* reverse	Mouse	ATAGCAAATCGGCTGACGGT
IL-6 forward	Mouse	ACAAAGCCAGAGTCCTTCAGAG
IL-6 reverse	Mouse	TGTGACTCCAGCTTATCTCTTGG
IL-1*β* forward	Mouse	TGCCACCTTTTGACAGTGATG
IL-1*β* reverse	Mouse	AAGGTCCACGGGAAAGACAC
GAPDH forward	Mouse	CCCTTAAGAGGGATGCTGCC
GAPDH reverse	Mouse	TACGGCCAAATCCGTTCACA
TNF-*α* forward	Rat	ATGGGCTCCCTCTCATCAGT
TNF-*α* reverse	Rat	TGCTTGGTGGTTTGCTACGA
IL-6 forward	Rat	AGCGATGATGCACTGTCAGA
IL-6 reverse	Rat	GGAACTCCAGAAGACCAGAGC
IL-1*β* forward	Rat	GACTTCACCATGGAACCCGT
IL-1*β* reverse	Rat	CAGGGAGGGAAACACACGTT
GAPDH forward	Rat	AGTGCCAGCCTCGTCTCATA
GAPDH reverse	Rat	GATGGTGATGGGTTTCCCGT

## Data Availability

All the data used to support the findings of this study are included within the article.

## Abbreviations

UC: Ulcerative colitis
DHA: Dihydroartemisinin
DSS: Dextran sulfate sodium.
